# Epidemiology and Factors Affecting Functional Outcome of Distal Radial Fracture in an Urban Tertiary Medical Centre in Malaysia

**DOI:** 10.5704/MOJ.2111.013

**Published:** 2021-11

**Authors:** WQ Chao, MZ Azman, SA Rosdi, TAW Tuan-Mustafa, YJ Tan, S Abdullah, AN Aizuddin

**Affiliations:** Department of Orthopaedics and Traumatology, Universiti Kebangsaan Malaysia, Kuala Lumpur, Malaysia

**Keywords:** radius fractures, emergency medicine, wrist, conservative treatment, non-surgical treatment

## Abstract

**Introduction:**

Distal radial fracture is a commonly encountered fracture. This study aims to study the epidemiology of distal radial fracture and factors affecting the patients’ functional outcome one to two years after the injury.

**Materials and methods:**

This is a retrospective cohort study. The records of patients, fulfilling the radiographical diagnosis of distal radial fracture, and aged 18 and above, who presented to our Emergency Department from 1st January 2018 to 31st December 2018 were retrieved. According to AO classification, we grouped our patients into A (extra-articular), B (partial articular) and C (complete articular). Patients with congenital abnormalities were excluded. Epidemiological data and relevant medical history were obtained and tabulated. A Malaysian language translation of Disability of the Arm, Shoulder and Hand (DASH) questionnaire was used to assess the functional outcome.

**Results:**

Out of 168 patients’ data retrieved, only 110 patients’ data were found complete for purposes of this study. The mean DASH score was 13.7 ± 7.87 approximately one to two years post-injury regardless of treatment method. Increasing age was associated with higher DASH score with r=0.407(p<0.001). Several variables had significantly better functional outcome: male gender (p=0.01), Type A fracture configuration (p=0.007) and non-operational treatment (p=0.03). There was no significant difference between treatment modalities in Type A fracture (p=0.094), but Type B (p=0.043) and Type C (p=0.007) had better outcome without surgery. There was no significant difference between different ethnic groups, open or closed fracture and mechanism of injury.

**Conclusion:**

Better functional outcome after sustaining distal radial fracture was associated with young age, male gender, type A fracture and treated non-operatively. Interestingly, more complex fracture pattern had better functionality were observed without surgery.

## Introduction

Distal radial fracture is a commonly encountered fracture in medical practice. The treatment and management of the fracture presents a substantial cost to a country’s healthcare sector. At the same time, sustaining the fracture would affect the quality of life of the patients, especially in the elderly. Several factors such as age^1-3^, gender^1-4^, mechanism of injury, type of fracture^5,6^ and treatment modalities^5-8^ have been recognised as factors affecting functional outcome. As such, a comprehensive epidemiological study would be useful in preventive measures for the injury and better management and the assessment of the outcomes.

Fracture patterns and demographic patterns vary in different regions. Several epidemiological studies of the injury and the ensuing functional outcome had been conducted in the countries such as Iceland^9^ and United States^10^, but limited articles were available in South East Asia, particularly Malaysia.

This study investigated the epidemiological pattern of the fracture, and was useful in understanding the pattern of the pathology. Besides, evaluation of the functional outcome of the patients using the Disability of the Arm, Shoulder and Hand (DASH) questionnaire one year to two-year following the injury could assist in identifying the factors affecting the recovery.

## Materials and Methods

**Fig 1:**
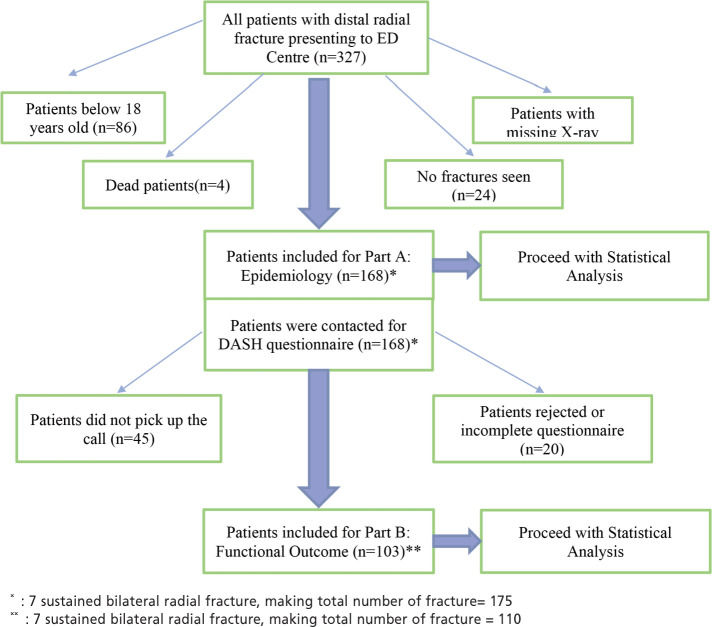
Shows the selection of the participants for the research.

This study design is retrospective cohort and included all 327 patients presenting to the Emergency Department from January 2018 to December 2018 with initial diagnosis of distal radius fractures. The hospital is a tertiary medical centre. In 2018, 75,684 patients presented to the Emergency Department (Fig. 1).

The Arbeitsgemeinschaft für Osteosynthesefragen/ Orthopaedic Trauma Association (AO/OTA) system was used to classify the fracture. Electronic and written reports were reviewed retrospectively. We included all patients aged 18 and above. Radiographs of the hand and forearm were reviewed to confirm the diagnosis of distal radial fracture. Patients with missing radiographs, with congenital abnormalities and those who refused to take part in the research were excluded from the study. Epidemiological data and related medical information were tabulated and analysed using SPSS version 27.0.

Disability of the Arm, Shoulder and Hand (DASH) questionnaire which is the most used questionnaire for musculoskeletal disorders across the entire upper extremity, was chosen as the instrument to assess the functional outcome of the patients one to two years after sustaining injury. DASH score varies from 0 to 100 points. The lower the score, the less disabled a person is, indicating better outcomes, and vice versa. In our study, we used a translated version of DASH questionnaire in Bahasa Malaysia (Malaysia Language) to aid in better understanding by the patient. The patient was contacted by our researchers by using mobile phone to conduct the interview for assessment of their functional outcome. All the scores were recorded and tabulated in SPSS software.

The mean DASH score was compared between the different groups of the factors at confidence interval of 95%. One-way ANOVA and Post HOC (Tukey test) were used to compare mean DASH score between three or more categorical data (AO classification of fracture). Mann-Whitney test was used to compare mean DASH score between two non-parametric data (gender, type of fracture and treatment modalities. Kruskall-Wallis were used to compare mean DASH score between three or more categorical data (ethnicity, mechanism of injury). Pearson correlation was used to investigate the relationship between age and DASH score. Significance was set at p<0.05. Ethics approval was obtained from the Research Ethics Board of Medical Sciences at our centre.

## Results

From January 2018 to December 2018, 75687 patients presented to the Emergency Department. Three hundred and twenty-seven patients were reported to have distal radius fracture. The records of 86 patients below 18 years old, four deceased patients, 45 with missing radiographs or missing information, and 24 patients with no fracture seen after reviewing the radiographs, were excluded. The number of patients fulfilling the inclusion criteria was 168, with seven of them sustaining bilateral distal radial fractures, which gave the total number of fractures investigated as 175.

By ethnicity, 76 (43.4%) were Malay, 66 (37.7%) were Chinese, 21 (12%) were Indian and 12 (6.9%) others. The mean age of our study patients was 54.2±20.78, with the youngest at 18 years and eldest 93 years. Ninety-six (54.9%) were male were and 79 (45.1) female, (M:F= 1.21:1). For the epidemiological study, the patients were separated into five age groups: (a) 18-20, (b) 20-29, (c) 30-39, (d) 40-49, (e) 50-59 and (f) 60-69. A peak incidence was seen in the age group of 60-69 years.

Males recorded highest incidence of distal radial fracture at the age group of 20-29 years old whilst females had the highest incidence at the age group of 60-69 years old (menopausal). The mechanism of injury was classified into (a) motor vehicle accident (b) fall (c) sports injury and (d) others. Our study reported a total of 63 cases of distal radial fracture due to MVA, 104 due to fall, 5 due to sport injuries and 3 other causes.

Out of 175 fractures sustained, 12 were open fracture while 163 were closed fracture. According to the AO classification, we grouped our patients into A (extra-articular), B (partial articular) and C (complete articular). Based on the interpretation on hand and forearm radiographs, most of the patients (n=80) of them had Type A fracture, followed by Type C (n=78) and Type B (n=17).

With regards to the type of treatment received, we grouped our patients into non-surgical and surgical intervention. Non-surgical treatment is defined as closed manipulation and reduction, immobilisation of joints with application of cast or splint. Surgical intervention is defined as surgery in the operating theatre involving open reduction and internal fixation of the fracture. Based on our findings, a total of 123 (70.3%) of them underwent non-surgical management and 52 (29.7%) underwent surgery.

From the data, 33 out of 63 (52.3% patients who had MVA sustained Type C (complete articular). In those with falls, 55.7 % out of 104 patients had sustained Type A fracture. We cross-tabulated the data of treatment modalities with respect to type of fracture. The result revealed that surgery was more likely to be done in Type B (41.2% [7 of 17 cases]) and C fractures (41.0% [32 out of 78 cases]), than Type A (16.2%) (Table I).

**Table I:** Shows the breakdown of treatment modalities of the patients with respect to the type of fracture

Type of Fracture		Treatment Modalities		Total
		Non-surgical	Surgery	
A	n	67	13	80
	%	83.8%	16.2%	100%
B	n	10	7	17
	%	58.8%	41.2%	100%
C	n	46	32	78
	%	59.0%	41.0%	100%

Surgery was more likely to be done for distal radial fractures in the younger age group, as compared to the elderly group.

Sixty percent of the patients from age group of 20-29 years had surgical intervention for their fractures. A decreasing trend was observed that as the age increases, fewer patients underwent surgery.

We contacted all of them for evaluation of functional outcome. Out of 168 patients, we managed to reach 130 of them via phone, 17 of them rejected the phone interview and three of them had i ncomplete DASH questionnaire filled with 3 missing questions. Therefore, 110 of the patients were evaluated for disability in the second part of the research. Comparison between different variables with respect to the DASH scores was done (Table II).

**Table II:** Comparison between different variable with respect to the DASH scores

Factors	Groups	n	Mean Rank	Mean DASH score	p value
Gender	Male	61	48.7	11.7 ± 7.5	p=0.01*
	Female	49	63.6	16.2 ± 7.7	
Ethnicity	Malay	59	54.7	12.6 ± 6.9	p=0.678
	Chinese	36	57.6	15.9 ± 8.1	
	Indian	7	43.1	17.0 ± 12.8	
	Others	8	60.8	9.2 ± 5.8	
Type of fracture:	Open	10	69.9	17.3 ± 7.7	p=0.135
	Closed	100	54.1	13.4 ± 7.8	
Type of fracture	AO Type A	45	-	11.3 ± 8.0	p=0.007*
	AO Type B	9	-	11.9 ± 3.8	
	AO Type C	56	-	16.0 ± 7.6	
Treatment Modalities	Conservative	75	51.2	11.8 ± 7.4	p=0.03*
	Surgery	35	65.2	17.8 ± 7.4	
Mechanism of Injury	MVA	43	50.2	13.0 ± 7.9	p=0.271
	Fall	63	59.9	14.2 ± 8.0	
	Sport Injury	2	30.5	17.1 ± 6.5	
	Others	2	72.0	11.3 ± 7.9	

*: p<0.05, there is significant difference between the groups

The DASH questionnaire was used to estimate the functional outcome of the patients after treatment at the hospital. The mean DASH score in our study groups was 13.7±7.9. Males had a mean DASH score of 11.7 which was found to be significantly lower than females with a mean DASH score of 16.2 (confident interval of 95%).

Our study also calculated the DASH score with respect to the different ethnicities, namely Malay, Chinese, Indian and Others. The Other ethnic group had better functional outcome than Malay, Chinese or Indians. However, the differences were not statistically significant.

**Fig 2:**
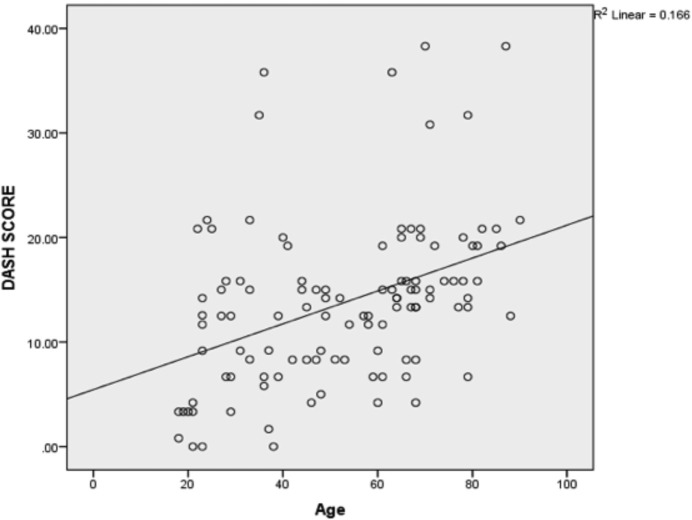
Shows the correlation between age and DASH Score. It shows Pearson coefficient = 0.407, r^2^= 0.166. There is weak positive correlation between DASH score and age of patients.

We cross-tabulated the DASH score against the age of the patients. It was found that there was a weak positive correlation between the two data, at the Pearson correlation of r=0.407 (Fig. 2). It meant that as the age of the patient increased, there would be increased disability of the patient after sustaining distal radial fractures.

For the breakdown by types of fracture, patients with closed fracture had a better DASH score than open type of fracture (p=0.135). For type of fracture according to AO classification, patients with Type C (complete articular fracture) had worst functional outcome than Types A and B fracture with a mean of 16.0, as compared to 11.3 of type A and 11.9 of type B. One-way ANOVA and Post HOC test had showed significant differences between Type A and Type C fractures.

Seventy-five of the patients underwent non-surgical management and the remainder underwent surgery. From our data, it was found that non-surgical treatment yielded better functional outcome as compared to surgery, with a mean difference of 5.9, at the significant level of p=0.03. We also further compared the mode of treatment for patients with different types of fracture under AO classification (Table III). As a result, we discovered that non-surgical management yielded better outcomes than surgical treatment in all type of fractures. However, comparisons in Type B and Type C yielded statistically significant result (p= 0.054 and p=0.007) whereas Type A were not significant.

**Table III:** Comparison of DASH score between the different treatment modalities in different types of fracture

AO classification	Treatment	n	Mean DASH score	p value
A	Non-surgical	36	10.3 ± 7.7	p=0.094
	Surgery	9	15.3 ± 8.4	
B	Non-surgical	5	9.7 ± 2.4	p=0.043*
	Surgery	4	14.6 ± 3.6	
C	Non-surgical Surgery	34	13.8 ± 7.1	p=0.007*
		22	19.3 ± 7.4	

*: p<0.05, there is significant difference between the groups

As to mechanism of injury, it was found that there was no significant difference in term of disability amongst the groups.

## Discussion

Literature from the Western hemisphere countries show a preponderance of females, especially in the peri-menopausal age group, being affected more than men. Women had a significantly higher proportion of radius/ulna fractures than men in the United States in 199810. The statement was further supported by Brogren *et al* and Sigurdarfottir *et al* who reported female to male ratio of 3.3:1 and 2.18:1, respectively in Sweden and Ireland^11,9^.

It should be noted that the social demography in Malaysia as a developing nation differs considerably to the developed nations. In our research, we noted that males outnumbered female with the ratio of 1.21:1 in direct contrast to literature from the Western hemisphere countries. Singapore shares a similar demographic and socioeconomic pattern as Malaysia and the ratio of males to females was 1.3:1. The article postulated that the pattern could be attributed to the high motor vehicle accident rate and the occupational-related injuries in Singapore^12^. The explanation would be highly relevant in Malaysia, as we had greater severity and higher incidence of motor-vehicle accident in Malaysia. According to WHO Global Status Report on Road Safety 2018, Malaysia had an estimated road traffic accident death rate of 23.6 per 100 000 population, as compared to 2.8 in Singapore^13^.

Over a 30-year period in North America (Rochester) between 1945 - 1974, the peak incidence of distal radial fracture was recorded in women in the age group of 60-64 years. There were no significant changes in the incidence of men in all age group and it was generally low as compared to women^14^.

A comparative study in Iceland between 1985 and 2004 observed different scenario in which there is an increase in the age-specific fracture in both males and females over the age of 60 years old9. In Sweden in 2004-2010, a bimodal pattern was observed for the age-related incident for distal radial fractures. For women, the first peak was seen at the age of 11 and the second peak was seen after 80 years of age. For men, the first peak was seen at the age of 13 and the second peak after 80 years old too^15^.

In contrast, the peak incidence of distal radial fracture was seen at the age of 50-60 years old in Singapore, earlier than the previous studies^12^. We recorded a different pattern of incidence where the peak was observed in age group of 20-29 years old for male, and 60-69 years old was the peak period where a female sustained distal radial fracture. This phenomenon can be explained by the higher incidence of MVA in young males (77.78% or 49 of 63 cases of MVA) and low bone mineral density in post-menopausal women.

For the assessment of functional outcome, we chose the Disability of Arm, Shoulder and Hand (DASH) questionnaire as a tool to measure the functional outcome. There are several methods of estimating disability following injury: namely Sarmiento radiological score, Gartland and Werley score, Green and O’Brien grade. However, we selected DASH as our instrument as it can detect and differentiate small and large changes of disability over time in patients with upper-extremity musculoskeletal disorders^16^. It is a patient-oriented and a subjective evaluation of functionalities, allowing us to understand the extent, and particularly the function of hand that is affected via the 30 questions covered in the questionnaire. A standardised measurement would permit comparison across various groups of patients or treatments, and is valuable for clinical research and can also meet the needs of government agencies and third-party payers for a means of assessing the relative impact of various conditions^17^.

Some studies focus only on certain groups of patients such as elderly patients above 70 years old^18^; and conservatively treated patients with intra-articular fractures^19^. However, we have selected all patients above 18 years of age with a diagnosis of distal radius fractures as we believe that this will allow us to better understand the factors affecting functional outcome for the general population such fracture.

Females were found to have poorer functional outcome than males, evidenced by a higher mean DASH score (p=0.01) in our study group. The result would perhaps suggest that males had better bone healing potential as compared to female. Loss of estrogenic protective factors particularly for post-menopausal women could be another explanation for the finding. In a retrospective study by Amorosa *et al*, their result was similar with ours where females had a significantly higher DASH score compared to males^18^. However, it must be noted that the mean age of their research group was 78 years old compared to ours of 54 years old.

In our study, we found that increasing age correlated with decreased functional outcome of distal radial fracture. As for the outcome of the treatment, aging had been well determined as one of the main factors that predict the affect the bone healing process, throughout different stages of bone fracture healing: in inflammatory regulation, cellular differentiation and signalling cascade^20^.

For the mechanism of sustaining injury, we classified the patients into MVA, fall, sport injuries and others. There is no significant association seen between the mechanism of injury and DASH score in our study. Due to limited information, we were not able to classify the type of falls. Ideally, we should classify the injuries according to the energy forces in trauma, as seen in other studies, for better understanding of the fracture mechanism. It would allow better correlation between mechanism of injury and the type of fracture, especially in the peri-menopausal age group thereby determining the appropriate treatment for the patient.

Perhaps the most important and interesting point in our study is that surgically treated patients appeared to have a worse outcome functionally. This is similar to the results of Barai *et al* but opposite to the results of Amorosa *et al*, who studied 58 patients and reported no significant difference between the non-operative and operative management^5,18^. As contrary to common belief among orthopaedic surgeons and previous studies that surgical intervention is preferred for a complex pattern of fracture as unstable fracture requires earlier stabilisation and immobilisation, we find that conservative management yielded better outcome than surgical treatment in all type of fractures. Comparisons in Type B and Type C yield statistically significant result (p= 0.054 and p=0.007). We postulate that there are several other factors such as lack of proper post-operative care, incompliance to post-operative physiotherapy and default follow-up would greatly affect the healing. Therefore, a future study can be done to observe the correlation of patient’s factors and functional outcome for surgically treated patients, so that a better candidate for surgery can be determined by the surgeon. A better post-operative care can also be planned to improve the recovery of the patient after the injury.

A limitation of this study is that the calculation of DASH scores post-injury was not able to detect the changes in capabilities of the patient in terms of upper limb or hand functionality. Our calculation only estimated the current disability, assuming that the patients’ initial DASH score was 0, in other word, assuming there was no impairment at all which is unlikely as thre there may be some disabilities, such as due to osteoarthritis or rheumatoid arthritis especially in the elderly, which were not excluded as the cause of the current disability after injury or treatment.

Further, we did not calculate the incidence rate as we believed that it was not statistically accurate to generalise our study sample to the whole Malaysian population. This is because our centre serves as a tertiary medical centre in a city, instead of a regional state hospital where the population is more clearly defined. Therefore, a multi-centres epidemiological study could be conducted in the future for better understanding of this aspect.

Finally, the sampling population only covered one year, 2018; therefore, a change in the epidemiological pattern could not be appreciated, and this may be rectified by a study with a longer duration.

## Conclusion

Better functional outcome after sustaining distal radial fracture was associated with young age, male gender, Type A fracture and treated conservatively. Interestingly, more complex fracture pattern had better functionality without surgery.
